# Expanding Access to Care: Qualitative Insights from a Nationwide Home-Based Test-to-Treat Program for COVID-19 and Influenza

**DOI:** 10.21203/rs.3.rs-8544556/v1

**Published:** 2026-01-09

**Authors:** Deogwoon Kim, Apurv Soni, Andrew Weitz, Kathleen Mazor, Kimberly Fisher

**Affiliations:** 1 UMass Chan Medical School; 2 National Institute of Biomedical Imaging and Bioengineering (NIBIB), National Institutes of Health (NIH)

**Keywords:** Telehealth, test-to-treat, home-based care model, COVID-19, influenza

## Abstract

**Introduction:**

The COVID-19 pandemic and subsequent influenza outbreaks highlighted disparities in timely access to tests and treatments. To address this gap, a nationwide Home Test to Treat (HTTT) program was launched to provide home test kits, telehealth consultations, and medication delivery for COVID-19 and influenza. This study explored participant experiences, factors influencing satisfaction levels, and recommendations for future programs.

**Methods:**

In-depth interviews were conducted with 48 participants enrolled in the HTTT program. Purposive sampling was used to obtain experiences from diverse backgrounds. Content analysis was used to extract the final coding scheme.

**Results:**

Interviewees reported a range of experiences, from positive to negative. Many of them were satisfied with efficient communication with telehealth providers, timely and convenient access to resources, and a seamless transition from enrollment to prescription. However, some interviewees noted limited interactions with telehealth providers, delayed access to treatment and cost challenges, and navigation and coordination challenges. For a future home-based Teat to Treat program, interviewees recommended improving inclusivity, offering more comprehensive consultation, enhancing user-friendliness, and increasing awareness through diverse platforms

**Discussion:**

This study highlights a home-based Test to Treat program as a feasible way to improve access to COVID-19 and influenza care. Enhancing interactions with providers, comprehensive care, and support for marginalized populations may further expand the program and reduce disparities in access to tests and treatments.

## Introduction

The COVID-19 pandemic underscored the global vulnerability to highly transmissible infectious diseases. It caused 5.73 million deaths during the first 18 months following the declaration of COVID-19 as a global health emergency in 2020 and became the third leading cause of death in 2021.^1^ Influenza rates dropped during the COVID-19 pandemic, likely due to decreased interpersonal contacts and greater adherence to preventive measures for respiratory infections. However, influenza cases have surged with declining COVID-19 mortality. The number of influenza-related deaths increased from 6,300 in the 2021–2022 season to 28,000 in the 2023–2024 season and 38,000 in the 2024–2025 season.^2^

Oral antiviral treatments, such as nirmatrelvir/ritonavir and oseltamivir, can significantly reduce the duration and severity associated with COVID-19 and influenza, respectively. Both treatments require prompt action for optimal efficacy: nirmatrelvir/ritonavir should be initiated within five days of symptom onset and oseltamivir within two days.^3,4^ The need to initiate treatment quickly may pose challenges for some patients, as individuals of low socioeconomic status or racial/ethnic minorities and residents in rural or high social vulnerability index communities are less likely to access testing and treatment,^5–8^ exacerbating health disparities.^9,10^

Brick-and-mortar test-to-treat programs gained attention during the COVID-19 pandemic as a way to improve access to timely testing and treatment for COVID-19. The programs provided rapid COVID-19 tests, prescriptions, and readily available medication in a single visit to increase efficiency and ensure timely treatment.^11^ However, these programs have largely been established in large metropolitan areas, covering only 23% of rural populations.^8^ To overcome this barrier, the Home Test-to-Treat (HTTT) program was launched to provide home test kits, telehealth consultations, and medication delivery for COVID-19 nationwide from August 15, 2023, to April 17, 2024. The program was designed to make COVID-19 testing and treatment for underserved populations and individuals with logistical barriers to traditional healthcare services. The initiative expanded to include influenza services on December 4, 2023, following the availability of at-home multiplex tests for both infections. This qualitative study aimed to describe the experiences of HTTT program participants, explore factors influencing program utilization and participant satisfaction, and identify recommendations for future programs.

## Methods

### Description of the HTTT Program

Information about the HTTT program was disseminated nationwide through offline and online advertisements, community outreach, and referrals. English or Spanish-speaking adults with an email address who were interested in the program registered via the HTTT website (www.test2treat.org). During registration, participants provided their medical history, current medications, and preferred telehealth modes. COVID-19 and influenza self-test kits were mailed to high-risk asymptomatic participants (aged over 65 years or with a chronic health condition) at the time of enrollment. Others could request testing if symptoms developed. Those who reported a positive for COVID-19 or influenza were offered a telehealth consultation and, if eligible, treatment. Consultations could occur via text, email, phone, or video, depending on the participant’s preference, availability, and state-specific regulations. Prescriptions for nirmatrelvir/ritonavir, molnupiravir, or oseltamivir were mailed to the patient’s pharmacy or home based on their preference. All services were initially provided at no cost; however, after the government subsidy for COVID-19 treatment ended, nirmatrelvir/ritonavir and molnupiravir were charged when picked up at pharmacies.

Semi-structured interviews were conducted to consented participants to explore their experiences and perspectives regarding the program. This study was approved by the WIRB-Copernicus Group (WCG IRB).

### Sampling and Recruitment for Interviews

We used a purposive sampling strategy to identify HTTT program participants diverse in race/ethnicity, sex, region, healthcare access, confidence in receiving healthcare, and experiences with the program. Potential participants were invited via email, with follow-ups call to non-responders. Telephone or video interviews were scheduled for those who expressed their interest. A $50 gift card was emailed after the interview as compensation for their time.

### Data Collection

Interviews were conducted between August 2023 and May 2024 and recorded with participants’ permission. In one instance, the recording failed due to a technical issue; in this instance, we used the interviewer’s notes for analysis. All interviews were conducted by a trained interviewer (DK).

The interviewer began by reviewing the purpose of the interview and confirming informed consent. The interviewer then explored interviewees’ first impressions of the program and delved into their experiences from enrollment through medication delivery. If interviewees did not utilize telehealth consultation and treatment, hypothetical questions were asked after briefly explaining what they could have experienced with these services. The interviewer asked probing questions as appropriate to understand the reasons behind their sentiments.

### Data Analysis

Recordings were transcribed by a professional transcriber and transferred to Microsoft Excel for analysis. We used a conventional content analysis.^12^ The interdisciplinary qualitative research team (DK, KF, KM) read a subset of transcripts to identify potential themes and develop a coding framework. The framework was iteratively revised based on discussion until the team reached a consensus on the final framework. One investigator (DK) coded all transcripts using the final coding scheme, and at least one other investigator (KM or KF) reviewed the results. Coding discrepancies were resolved through discussion with the qualitative team.

## Results

### Interviewee Characteristics

Interviews were conducted with 48 interviewees. More than half were aged 31–50 years, self-identified as female, non-Hispanic White, and held college or postgraduate degrees. Approximately 46% of interviewees lived in the Northeast at the time of enrollment. Thirty-three percent of the interviewees had no insurance, and 25% had self- or employer-sponsored insurance (Table 1).

### Reasons for Program Participation

Interviewees reported positive initial impressions when they first learned about the program. They valued the free, easy, and quick access to the COVID-19 and influenza self-test kits, consultations, and treatment. The access was especially beneficial for individuals without insurance or a primary care provider. For interviewees without symptoms at the time of enrollment, the program served as their backup plan to access free tests and treatment. For those who enrolled immediately after testing positive for COVID-19 or influenza, the availability of a telehealth consultation was attractive, as it provided access to a clinician and potentially treatment outside of regular office hours without leaving home, which was particularly desirable when feeling unwell.

However, several interviewees reported initial hesitation toward the program. Some interviewees felt “it sounded too good to be true” and suspected that it might be a scam. Others doubted its logistics and the quality of the consultations it would provide. Concerns about sharing personal information were also mentioned. Some felt reassured after learning the program was affiliated with a government entity and a public university-affiliated hospital, was supported by a prominent physician and public health researcher, or found positive testimonials on multiple online platforms. An explicit disclaimer about privacy protection alleviated concerns for others.

Interviewees ultimately decided to join the program because they believed that the benefits, such as quick access to resources and convenience, outweighed any potential drawbacks. Quotes representing each theme are listed in Table 2.

### Program Experiences

#### Positive Experiences

Many interviewees reported that the anticipated benefits were realized: they accessed the desired services and treatment when they needed them without significant challenges.

##### Efficient communication with telehealth providers.

Many interviewees, particularly those who joined the program specifically for treatment, expressed satisfaction with their communication with the telehealth providers, which they described as “very efficient, and to the point, and straightforward.” Providers’ confidence, comprehensive instructions on treatment, and warm wrap-up messages increased interviewees’ satisfaction. Trust in the program also increased interviewees’ trust in providers.

##### Timely and convenient access to resources.

Many interviewees who joined the program to access treatment were aware of the time-sensitive nature of the treatment window for COVID-19 and influenza before enrollment and sought to receive medications as quickly as possible. They were satisfied that the program provided telehealth consultations and prescriptions outside regular clinical hours or when their primary care provider was unavailable, including during vacations. Most interviewees who were prescribed medication received it promptly, either through their local pharmacy or mail delivery, allowing them to start medication within the critical treatment window.

Additionally, receiving a telehealth consultation at home was seen as advantageous. It eliminated the need to dress up, drive, or spend time waiting, and avoided exposing others to viruses. Interviewees had the option to have medication delivered directly to their homes, which further increased the convenience of the program.

##### Seamless transition from enrollment to prescription.

Most interviewees reported that the overall process from enrollment to prescription was easy, simple, smooth, and streamlined. Prompts were placed appropriately throughout the process and helped participants navigate seamlessly across different platforms.

#### Negative Experiences

Despite the many positive experiences reported, some interviewees still had negative experiences with the program.

##### Limited interaction with telehealth providers.

Although most interviewees acknowledged that minimal interaction with telehealth providers reduced hassles and facilitated quick access to treatment, some interviewees still preferred to have more reciprocal interaction to discuss their health conditions and treatment options in greater detail. For example, an interviewee who was prescribed nirmatrelvir/ritonavir and was taking a statin researched potential drug interactions and felt that the exchange of information with the provider was insufficient.

The singular episodic nature of the relationship between participants and telehealth providers also limited opportunities for follow-ups or addressing subsequent questions. The lack of continuity in care particularly affected patients on multiple medications who required monitoring after treatment initiation. For instance, during the telehealth consultation, one COVID-19-positive interviewee who was taking an anticoagulant and a statin presented supporting evidence from an NIH website and confirmation from his anticoagulant specialist that nirmatrelvir/ritonavir could be used safely with closer International Normalized Ratio (INR) level monitoring. However, the telehealth provider ultimately declined to prescribe nirmatrelvir/ritonavir even after this detailed information, as he could not provide necessary follow-up care.

Interviewees who wished to have a more traditional form of consultation preferred a video telehealth visit. However, some eventually opted for email communication, despite their initial preference, after being informed that it would be the fastest option, as their priority was quick access to treatment.

##### Delayed access to treatment and cost challenges.

Several interviewees missed the critical treatment window for nirmatrelvir/ritonavir or oseltamivir due to delays between enrollment and telehealth visits. Additionally, providers’ delayed email responses further prolonged prescription times, especially when multiple rounds of email communication were needed between providers and interviewees.

A few interviewees reported that their prescription was unavailable at their local pharmacy (contrary to what they had been told to expect). Others found that while the medication was available at their local pharmacy, it was not free. During program implementation, the federal government subsidy for COVID-19 treatment ended, resulting in nirmatrelvir/ritonavir no longer being offered for free from the program when picked up at pharmacies. However, two interviewees reported not being informed in advance of the cost and were confused when they were unexpectedly charged at a pharmacy.

##### Navigation and coordination challenges.

For some interviewees, the process did not unfold as smoothly as expected. Navigating three different interfaces for enrollment, telehealth consultations, and medication delivery was cumbersome for those with limited technological skills. Engagement with three different interfaces also caused confusion about identifying the appropriate platform for follow-up communication.

Two interviewees experienced a disruption when sending their prescriptions to local pharmacies, resulting in delays in receiving their medications or, in one instance, the complete loss of the prescription.

#### Recommendations

##### Enhanced access for marginalized populations.

Interviewees recognized the value of this program in improving access to healthcare and expressed that it should be continued. Some hoped that it could be expanded to address other diseases as well. They highlighted the importance of reaching vulnerable populations, including non-English or non-Spanish speakers (the only two languages for which the program was available), older individuals with limited computer skills, people living in rural areas with limited Internet access, or recent immigrants.

##### Comprehensive and personalized consultation.

Several interviewees recommended that the program provide more comprehensive information, such as guidance on potential side effects or the expected course of illness after taking the medication. They believed this would help them assess whether their symptoms were within a normal range or required further attention. They also recommended more personalized, human-to-human interactions and follow-up care.

##### Improved user-friendliness.

Some interviewees suggested ways to improve the user interface, such as offering clearer guidance on the overall process and what to expect next. Other suggested improvements in website design, including record-keeping, a shorter dropdown list to reduce scrolling, and better integration across different platforms to enhance cohesiveness.

##### Sources to spread the information.

The majority of interviewees encouraged researchers to disseminate the program information widely to better reach people who might benefit from the program. They suggested diverse platforms for advertisement, such as social media, newspapers, or subway advertisements. Some suggested specific respected resources or trusted organizations as sites to disseminate the program information.

Table 3 presents recommendations for future HTTT programs based on interviewees’ experience and insights.

## Discussion

In our study, interviewees consistently praised the HTTT program’s convenience, rapid access to treatment, and decentralized, at-home care delivery. Many interviewees expressed high satisfaction with the ease of obtaining tests and quickly connecting to telehealth providers without typical geographic or logistical barriers. They valued the ability to get timely consultations and antivirals from home, avoiding clinic visits and delays. This strong endorsement of the program’s “test, telehealth, and treat at home” model underscores that making care easy and immediate was critical to its success. Participants especially appreciated free services and flexible clinic hours, which allowed them to receive care on their own schedule, and felt these features should be continued in future programs. These findings highlight that convenience and speed, coupled with minimal barriers, are paramount from the patient’s perspective in any home-based test-to-treat initiative.

The positive themes from our study align closely with prior research on telehealth and remote care, particularly around convenience and accessibility.^13,14^ During COVID-19 lockdowns in New Zealand, for example, patients found telehealth both convenient and effective due to the elimination of travel and time barriers.^15^ Broader HTTT data also support how home-based care can close geographic gaps; in the HTTT main cohort, 83% of those who tested positive used telehealth consultations, mostly outside normal business hours, illustrating the demand for care at all hours. Further, 82% of HTTT telehealth users received antiviral prescriptions, with nearly 60% treated within one day of symptom onset. This fast turnaround, which is critical for antiviral effectiveness, reflects interviewees’ description of the services as fast and lifesaving. Similar benefits have been reported beyond COVID-19, with telehealth improving patient engagement, outcomes, and even reducing costs in chronic disease management by overcoming geographic and access barriers.^16,17^ Such evidence reinforces that decentralized care models can benefit diverse patient populations, including rural and chronically ill patients, by bringing care to the patient instead of the patient to care. Public health programs leveraging technology have demonstrated similar success. For example, a Los Angeles County initiative delivered tens of thousands of test results and over a thousand antiviral prescriptions via a digital platform, predominantly to high-vulnerability communities.^18^ Our qualitative insights contribute to existing literature by showing that when remote care is user-centered and accessible, it drives high satisfaction and uptake, suggesting strong potential for broader application beyond the pandemic.

However, the interviews revealed a lack of comprehensive care as a key limitation of the program. While many interviewees found the consultations efficient, some interviewees taking multiple medications described the interactions as limited and insufficient. The absence of structured follow-up further compounded this concern, leaving these participants without a clear way to address post-consultation questions and, in some cases, preventing them from receiving a prescription due to the program’s limited capacity for necessary follow-up care. A lack of channels for post-consultation questions or side-effect management in the HTTT program underscores the need for continuity of care, even in an acute care model. Future implementations should integrate structured follow-up calls or messages, post-treatment check-ins, and clear referral pathways for patients requiring ongoing support or having complications.

Interviewees also identified several technical and usability barriers that could be improved. Participants reported difficulties navigating multiple interfaces and platforms during the process, indicating that the user interface was not seamless for everyone. Simple fixes like providing step-by-step guidance and live technical support can significantly improve user experience, particularly for individuals who are less comfortable with technology.^19^

Another related issue raised was the digital divide. Limited internet access among some elderly and rural participants underscored how technology-based services can unintentionally exclude certain groups.^20,21^ Quantitative data from the HTTT program supported this concern: older adults and Medicare beneficiaries were considerably less likely to engage in telehealth services in the program, suggesting that low digital literacy or preference for in-person care may have limited participation. Additionally, Black and Hispanic participants in the HTTT cohort experienced modest delays in obtaining treatment, highlighting persistent access barriers even when cost and distance were removed. These patterns mirror broader telehealth trends, where vulnerable populations may be marginalized due to limited device access, lower digital literacy, or trust issues, and where telemedicine, without deliberate efforts to promote equity, can exacerbate disparities.^22,23^

### Strengths and Limitations

Our study offers insights into future home-based test-to-treat programs by capturing a wide range of participant experiences across the country. However, our interviewees were predominantly non-Hispanic Whites, highly educated, and residents of the Northeast, and all interviews were conducted in English. Thus, findings may not fully reflect the experiences of non-English speakers, racial or ethnic minorities, individuals with lower education levels, or residents of other regions. Additionally, as our interviewees were HTTT program participants, findings do not capture the perspectives of those unaware of or did not participate in the program. Lastly, findings may not be generalizable to the home-based test-to-treat programs for chronic conditions, which require more integration with the healthcare system and longitudinal follow-ups.

## Conclusion

This study highlights a home-based test-to-treat program as a feasible approach for improving access to COVID-19 and influenza tests and treatment. Participants appreciated its convenience, efficiency, and timely access to telehealth consultations and treatment. However, limited interaction with providers and technical barriers reduced satisfaction for some users. More comprehensive consultation and additional support for technology and language for marginalized populations can help future programs better reach high-risk populations and promote equitable access to testing and treatment.

## Figures and Tables

**Table 1.** Interviewee Characteristics (N=48)

Characteristic	n (%)
Age(years)^a^	
18–30	3 (6.3%)
31–40	20 (41.7%)
41–50	12 (25.0%)
51–60	5 (10.4%)
61–70	4 (8.3%)
Greater than 70	4 (8.3%)
Sex^b^	
Female	25 (52.1%)
Male	22 (45.8%)
Non-binary	1 (2.1%)
Ethnicity/Race^b^	
Non-Hispanic White	25 (52.1%)
Non-Hispanic Asian	9 (18.8%)
Non-Hispanic Black or African	1 (2.1%)
American	
Hispanic	9 (18.7%)
Others	4 (8.3%)
Education^c^	
High school/GED	2 (4.2%)
Some college	4 (8.3%)
College graduate	9 (18.7%)
Postgraduate	18 (37.5%)
Refused	1 (2.1%)
Missing	14 (29.2%)
U.S. Census Region^a^	
Midwest	4 (8.3%)
Northeast	22 (45.8%)
South	12 (25.0%)
West	10 (20.9%)
Insurance Type^b^	
None	16 (33.4%)
Medicare	6 (12.5%)
Medicaid	6 (12.5%)
Private insurance	12 (25.0%)
Other	4 (8.3%)
Missing	4 (8.3%)

Source: ^:^

a Data collected from 48 self-reported surveys drawn from the main Home Test-to-Treat (HTTT) Program;

b Data collected from 48 in-depth interviews;

c Data from 48 participants obtained through surveys from the main HTTT Program and in-depth interviews

**Table 2.** Themes and Example Quotes on Participant Experience with the Nationwide Home Test-to-Treat (HTTT) Program

Themes	Subthemes	Quotes
**Reasons for Program Participation**		“I thought it was a great … I mean a great program to sign up; get tests; and care; and medication if you were eligible; all for free; all without having to leave your house. I mean … it’s great.” (ID 27) “What made me feel more comfortable was just knowing that it was recommended by Doctor [influential physician], and I could likely get Paxlovid. And from what I had read online, and Reddit, and Twitter, from the people that … other people in the community, that it was a good resource.” (ID 33)
**Program Experiences**		
Positive Experiences	Efficient communication with telehealth providers	“It was … very efficient. There was one email exchanged, which was the email I received after the fact saying my prescription … or submitted. […] It was very simple.” (ID 9) “She had all the information that I put in at hand. And just … asked a couple of questions, went over the medicine, very detailed with the instructions on how to take it.” (ID 19) “And then the other kind of sweet thing, […] after I had asked my question and then thanked him, and then he said, ‘Feel better soon.’ Now that seems really tiny, but it was actually … Oh, he knows I’m … there’s an actual person here. And that made me feel a little better.” (ID 28) “I think it [trust in telehealth providers] stems from the program. Like I trust the program; therefore, I trust the person.” (ID 44)
	Timely and convenient access to resources	“The only thing that I worried about was the speed of medication being delivered because I know that in order to have this full effectiveness of the medication, then a patient needs to take it within five days of symptoms. […] And to my surprise … it was very quickly … delivered.” (ID 44) “Well you know, when you’re sick, just getting a consultation and … it was actually … not having to be on camera when you’re really sick is a good thing. And not having to drive … I mean it would have been … I would have had to drive more than an hour to urgent care, and I was not in good shape to be driving.” (ID 28)
	Seamless transition from enrollment to prescription	“It surprisingly went smoothly. It’s like government things usually expect hiccups, but it was not that. It was very smooth.” (ID 48) “It didn’t ask for a lot of extraneous information. And it seemed that all of the prompts wanted to be helpful.” (ID 9)
Negative Experiences	Limited interaction with telehealth providers	“I suppose it would have maybe been better practice to talk … have a back-and-forth with a … between the doctor and the patient. Just to be like, ‘Do you know how to take this? Do you know not to take your Statin as well?’ […] I Googled that stuff on my own.” (ID 23) “So … one thing that I noticed though was the chat was not available after a couple of days, because that’s when I tried to get amoxicillin.” (ID 21) “The one thing that caught my attention was they said, ‘You can do it by phone, video conference, or email.’ And then there was a note on there that said, ‘Because of demand, or whatever, email is usually the fastest.’ So I thought, okay, well I’m going to do email because it’s most likely to get the fastest response.” (ID 34)
	Delayed access to treatment and cost challenges	“Theoretically, you wouldn’t have to wait; however, the telehealth experience I had was not very good. It took them four days to respond to that email, which means by the time they sent me the prescription, it was too late to start.” (ID 38)
	Navigation and coordination challenges	“So I didn’t know where to send my question [about the treatment window for oseltamivir]. The [telehealth] platform was a little confusing.” (ID 28) “I went to the pharmacy, and they happened to be closed that day, and so I think that’s the reason why the prescription was never filled or was not in their system. So I ended up never getting the prescription.” (ID 40)
Recommendations	More inclusivity	“It would have been nice to have an RSV test too, actually, because there’s been a lot of RSV going around in people my age in this part of New England. A strep test would have been great.” (ID 28) “My aunt is a recent immigrant from Venezuela five years ago, and she’s not recently a citizen. And she definitely struggles with access to medical care. And I didn’t know at the time that I was sick that she was also sick, and I wish that I had told her about the program.” (ID 33)
	Comprehensive and personalized consultation	“I would say like better response times and maybe if they could be more … they could go a little bit more in depth in detail. Because some people may not understand.” (ID 41) “I guess I could see how some people would appreciate somebody maybe having … more empathy or drawing that connection out with somebody; not to say that whoever was treating me was unempathetic, that’s not accurate, just efficient.” (ID 32) “Maybe a quick follow-up email […] just a quick check in to see, “Oh, did you have any difficulty? Are you okay?” That’s really it.” (ID 30)
	Improved user-friendliness	“So it’s like, “Okay, yeah, I can fill out my name and address on this and hit next.” And then now, “Oh, they want this. Okay. I can do that too and hit next.” […] But maybe an overview at some point would have been appreciated. […] you even have a little progress bar or steps on the side and you can see where you’re at in the set up.” (ID 32) “I think it’s so important to provide some sort of record-keeping function to the participants too so that they can see what they did and that they actually filled out the questionnaires.” (ID 4) “I felt like through the process there was a lot of times when I was going back and forth between email, and a chat window, and … other windows […] that may have felt a little disjointed. Where if it could all be combined, […] that could be to make something work cohesively and streamline it across various devices, and platforms, and operating systems.” (ID 32)
	Sources to spread the information	“It should be tied to our County Department of Health and make that something that they would put on their website, which is pretty extensive.” (ID 29) “So articles published in respectable resources, one of them being AARP Newsletter, something that I can … or the New York Times, Medicare communications.” (ID 34) “Public health agencies … they’re non-political agencies dedicated to public health telling you about these.” (ID 37)

**Table 3.** Recommendations for Future Home-Based Test-to-Treat Programs

**Continue to offer convenient and affordable self-tests, consultations, and treatments.** Ensure that these services remain accessible and free of charge to encourage widespread use. **Communicate clearly what is being offered.** If there are options for telehealth consultations (e.g., email vs. video) or medication delivery (e.g., mail delivery vs. pharmacy pickup), provide clear and explicit details of each option, including timing, costs, constraints. Ensure that services are delivered as promised. **Provide evidence of the program’s credibility.** Affiliation with respected organizations and/or businesses can build trust. Dissemination of program information through trusted and respected sources can enhance credibility. **Offer easy-to-use means of following up with a provider.** Ensure that participants can easily follow up with a provider to get their questions answered or receive assistance if there is a glitch in the process. **Provide additional support for non-tech savvy individuals.** Offer extra assistance to those who may not be familiar with technology to ensure they can access and benefit from the services.

## Data Availability

Transcripts will not be publicly shared to protect interviewees’ privacy and confidentiality. The codebook may be shared upon request through the corresponding author.
